# Sarcopenia and Anemia in Elderly Koreans: A Nationwide Population-Based Study

**DOI:** 10.3390/healthcare11172428

**Published:** 2023-08-30

**Authors:** Do-Youn Lee, Sunghoon Shin

**Affiliations:** 1 Research Institute of Human Ecology, Yeungnam University, Gyeongsan 38541, Republic of Korea; triptoyoun@yu.ac.kr; 2 Neuromuscular Control Laboratory, Yeungnam University, Gyeongsan 38541, Republic of Korea

**Keywords:** sarcopenia, anemia, elderly, low muscle mass, hemoglobin

## Abstract

Sarcopenia and anemia are common diseases in the elderly and are caused by various factors. In this study, the association between sarcopenia and anemia in an elderly Korean population was examined. The Korea Centers for Disease Control and Prevention’s cross-sectional, nationally representative Korea National Health and Nutrition Examination Survey (KNHANES, 2008–2011) served as the source of the data for this study. Of the 2769 participants (1167 men and 1602 women) included in this study, a significant association was found between sarcopenia and anemia in the elderly in Korea. In Model 1, unadjusted for covariates, the prevalence of sarcopenia in all participants was 1.805 (95% CI 1.364–2.388) and 2.746 (95% CI 1.740–4.334) in men, and 1.494 (95% CI 1.045–2.138) in women. In Model 4, adjusted for all covariates, the prevalence of sarcopenia in all participants was 1.455 (95% CI 1.064–1.989) and 2.649 (95% CI 1.475–4.755) in men, but it was insignificant in women. While prior studies failed to consider variables such as exercise status and nutritional intake, this research incorporated these factors as covariates. Despite this comprehensive approach, this study still revealed an independent association between sarcopenia and anemia. Moreover, a significant association was uncovered among elderly men, with no corresponding association identified among women.

## 1. Introduction

Although the mechanism of sarcopenia has not yet been identified, it is related to various factors such as lack of exercise, aging, and hormonal changes, and is a disease in which muscle mass, muscle strength, risk of falls, and physical performance decrease [1]. Weakness of muscle leads to decreased physical activity and restrictions on mobility [2,3], and decreased muscle mass is associated with osteoporosis, metabolic disorders, cardiovascular disease, physical damage, and high mortality [4,5,6,7]. Sarcopenia shows an increase in the risk of hospitalization, disability and mobility, and the risk of falls in the elderly [1,2,3]. Furthermore, it is caused by alterations in oxidative stress and chronic inflammation, neuromuscular aging, muscle protein turnover, hormone and cytokine imbalance, and behavioral and nutritional variables [8]. The pathophysiology of sarcopenia and the related treatments are further complicated by the intricate interplay between the aforementioned variables. Hemoglobin (Hb) has been identified as a key biomarker for sarcopenia in both diagnostic and prognostic dimensions, among all parameters associated with the development of sarcopenia [9].

Anemia or low Hb levels are a common disease in the elderly and is caused by a decrease in the oxygen supply owing to a lack of hemoglobin. It has several pathophysiological causes [10,11]. In addition, it not only lowers physical performance, physical function, and strength but also increases disability and mortality [12,13]. Anemia is a major cause of malnutrition and inflammation [14,15], and is associated with various chronic diseases [16,17,18,19]. Therefore, clinical follow-up is important if the hemoglobin level is lower than normal. In addition, although the association between anemia and unfavorable health outcomes in the elderly has been extensively shown, the operational definition of anemia in this age group is still debatable because it is influenced by sex, age, and ethnicity.

Sarcopenia and anemia are caused by various factors and some of the factors that cause anemia also affect the occurrence of sarcopenia. Muscle atrophy and oxidative stress are caused by inactivity, environmental factors, and aging [20,21], which are known to have a deleterious impact on blood hemoglobin concentration [22]. Sarcopenia and anemia are caused by aging and other factors; thus, studies considering these factors are needed.

Several studies have reported an association between sarcopenia and anemia. However, the patients are either limited to a specific sex [23,24,25] or those with a specific disease [26]. In addition, the correlation between the two conditions is still unclear because studies have shown that the relationship exists only in men or women [23,25,27]. Moreover, most studies have not considered factors affecting both conditions, such as lifestyle and nutritional factors [23,24,27,28]. As such, the roles of anemia in muscle function and muscle mass have been studied, but the associations remain unclear. Therefore, this study aimed to investigate the association between sarcopenia and anemia in the elderly by considering sex and influencing variables such as lifestyle, exercise status, and nutritional intake, using data from The Korea National Health and Nutrition Examination Survey (KNHANES).

## 2. Materials and Methods

### 2.1. Data Source and Sampling

**Figure 1:**
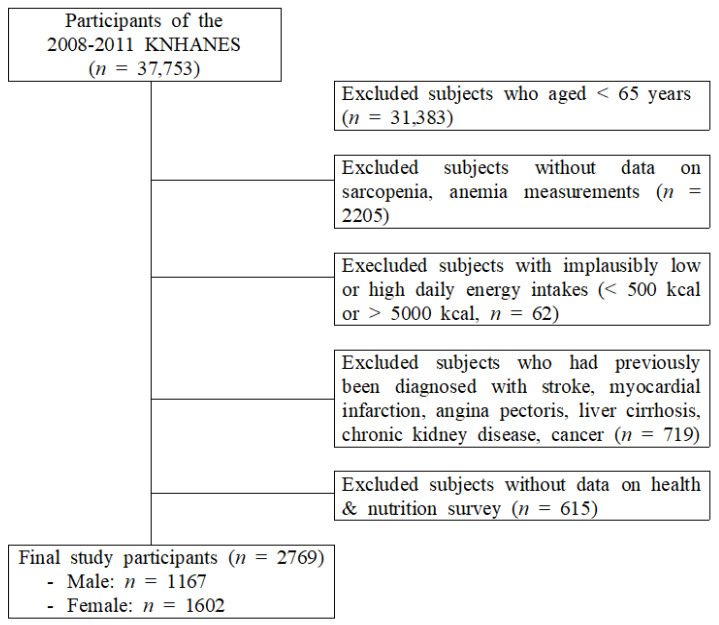
Selection of participants from the Korea National Health and Nutrition Examination Survey 2008–2011.

This study obtained data from the KNHANES (2008–2011), a cross-sectional, nationally representative survey conducted by the Korean Centers for Disease Control and Prevention. This study included adults aged over 65 years who underwent a whole-body dual-energy X-ray absorptiometry (DXA) and responded to examination and health surveys. Among the 37,753 participants in the KNHANES, 6370 aged ≥65 years were selected. The following participants were excluded: 2205 without sarcopenia and anemia measurements; 62 with implausibly low or high daily energy intake (<500 or >5000 kcal/day); 719 diagnosed with stroke, myocardial infarction, angina pectoris, liver cirrhosis, chronic kidney disease, or cancer; 615 who did not participate in the health and nutrition survey. Finally, 2769 participants were selected (Figure 1).

### 2.2. Measurements of Variables

#### 2.2.1. Covariates

Height, weight, systolic and diastolic blood pressure (SBP, DBP), triglyceride, fasting glucose, body mass index (BMI), total cholesterol, and high-density lipoprotein cholesterol (HDL-C) levels were measured during the physical examination. After a 10 min rest period, blood pressure was measured in a seated position using a mercury sphygmomanometer. All participants underwent two measurements taken at 5 min intervals. For data analysis, the average of two measurements was used. Waist circumference was measured during full expiration at the midpoint between the top of the lateral border of the iliac crest and the bottom of the rib cage. After overnight fasting, blood samples were taken in the morning and evaluated at the national central laboratory. Body mass index (BMI) was calculated by dividing weight (kg) by height (m^2^).

In order to classify the aforementioned variables into categories, they were classified by the following criteria: abdominal obesity (waist circumference ≥ 90 cm for men and ≥80 cm for women), high BP (systolic BP ≥ 130 mm Hg and/or diastolic BP ≥ 85 mm Hg), hypertriglyceridemia (≥150 mg/dL), low HDL-C level (<40 mg/dL for men and <50 mg/dL for women), or high fasting glucose level (≥100 mg/dL). Subjects who reported taking antihypertensive agents or hypoglycemic agents were considered to have high BP or a high fasting glucose level.

Education level was classified as lower than high school, high school, or higher than high school. Living with a spouse was classified as being “married”. Individual income levels were classified into quartiles. Smoking status was divided into three groups: current smokers, ex-smokers, and never-smokers. Alcohol consumption status was divided into two categories: current users and non-users. Resistance exercise frequency was assessed by the participants’ responses to the question, “How many times a week do you perform resistance exercise such as sit-ups, push-ups, or lifting dumbbells or barbells?” Participants’ current walking was measured using a short version of the International Physical Activity Questionnaire in Korea [29], which measures health-related physical activity in populations. The number of days in the previous week that the participants walked ≥10 min at a time was recorded. The total walking time (TWT) per week was calculated as follows:

TWT = walking days (days/week) × walking minutes (min/day).

Before the nutrient intake evaluation, all participants were instructed to continue their regular eating habits.

The daily intake of total energy, carbohydrates, proteins, fat, and iron was assessed. Daily food intake was measured using the 24 h recall method based on a weekday’s food consumption, in which all food content and consumed amounts during the last 24 h were obtained from the participants. Based on these data, consumed nutrients and electrolytes were calculated using the food composition table which was made and validated by Rural Development Administration. Dietary variables used in this study included total energy (kcal/day), carbohydrate (%energy), total fat (%energy), protein (%energy), fiber (g/1000 kcal), and daily nutrient intake was calculated using Can-Pro 2.0.

#### 2.2.2. Measurements of Sarcopenia

Licensed technicians used DXA (Discovery QDR 4500 W, Hologic Inc., Belford, MA, USA) to determine muscle mass and body composition. Participants fasted before the assessment and were placed in the supine position. Skeletal muscles were considered non-fat and non-bone tissues. Appendicular skeletal muscle mass (ASM) was calculated as the sum of skeletal muscle masses measured using DXA in both arms and legs. The skeletal muscle mass index (SMI) was calculated as ASM (kg) divided by height in meters squared (m^2^). Sarcopenia was defined as SMI < 7.0 kg/m^2^ for men and <5.4 kg/m^2^ for women, as recommended by the Asian Working Group for Sarcopenia [30].

#### 2.2.3. Measurements of Anemia

Blood analysis, including Hb level measurements, was performed and used as an index to determine the level of anemia. Hemoglobin levels were measured by the cyanide-free sodium lauryl sulfate method using XE-2100D (Sysmex, Kobe, Japan). Anemia was defined by the World Health Organization (WHO) as hemoglobin levels of <13 g/dL in men and <12 g/dL in women [31].

### 2.3. Data Analysis

The data were analyzed using SPSS 27 (IBM, Armonk, NY, USA). The analysis of the data was weighted with reference to a multistage, complex probability sampling design. Data are expressed as absolute numbers and estimated percentages with standard errors (SE). Categorical variables were expressed as both counts and percentages. Continuous variables were expressed as the mean ± SE. The χ^2^ test or Student’s *t*-test was used to evaluate the demographic and clinical characteristics differences by sarcopenia and anemia. Multivariate logistic regression analysis investigated the association between anemia and sarcopenia. Odds ratios (ORs) and 95% confidence intervals (CIs) were estimated using multiple logistic regression analysis. All covariates used in logistic regression analysis were input as categorical variables to solve the multicollinearity problem. Statistical significance was set at *p*-value < 0.05.

## 3. Results

Table 1 shows the characteristics of the participants according to anemia status and sex. Regardless of sex, there were significant differences in age, weight, height, BMI, total cholesterol, triglycerides, DBP, and waist circumference according to anemia status. In addition, there were significant differences in height, income level, low high-density lipoprotein cholesterol level, ASM, and SMI among men. In women, there were significant differences in ASM according to alcoholism, marital status, high-density lipoprotein cholesterol level, and anemia. There was no significant difference in smoking status, resistance and aerobic excise, high fasting glucose and blood pressure, vitamin D, energy intake (kcal), carbohydrate, protein, and iron intake according to anemia in both men and women.

Table 2 shows the characteristics of the participants according to sarcopenia status and sex. Regardless of sex, there were significant differences in age, weight, height, BMI, waist circumference, abdominal obesity, energy intake, and carbohydrate, protein, and fat intake. Significant differences in drinking status, income level, resistance exercise, aerobic exercise activity, HDL cholesterol level, and decreased HDL cholesterol levels were observed among men. However, women showed significant differences in smoking status, marital status, diastolic blood pressure, and iron consumption. There was no significant difference in high fasting glucose, glyceride, and blood pressure according to sarcopenia in both men and women.

Table 3 shows the association between sarcopenia and anemia. The prevalence of sarcopenia in Model 1 was 1.805-fold higher (95% CI 1.364–2.388) than those of non-anemia, 2.746 (95% CI 1.740–4.334) in men, and 1.494 (95% CI 1.045–2.138) in women. Model 4 showed an OR of 1.394 (95% CI, 1.016–1.915) for all participants after adjusting for all factors that potentially affected sarcopenia and anemia. When the OR was examined independently by sex, there was a significant association of 2.429 (95% CI, 1.309–4.505) in men but not women. OR tended to decrease as variables that could affect were adjusted, but there was still a significant association between sarcopenia and anemia.

## 4. Discussion

The purpose of this study was to determine the association between sarcopenia and anemia. The primary findings of this study were that sarcopenia and anemia were independently associated after adjusting for various covariates, such as personal lifestyle, smoking, drinking, nutritional status, and exercise. However, when this association analysis was divided by sex, men with anemia were more likely to develop sarcopenia, and their skeletal muscle mass was lower than that of women. In women, this was not significant.

The results of this study are consistent with those of previous studies. A previous study reported that the elderly with low Hb levels is more likely to develop sarcopenia and muscle weakness. These results were more pronounced in older men than in elderly women [32]. Another study reported that individuals with higher Hb levels have higher muscle area and density, less fat area, and decreased muscle strength, usually in the presence of anemia [22]. In addition, studies have reported that low hemoglobin levels in kidney transplant recipients are strongly associated with decreased muscle mass and strength [26].

The biological mechanisms underlying the association between sarcopenia and anemia can be explained in several ways. Hb transports oxygen to all organs and tissues in the body [33]. In this study, anemia was defined as a low Hb concentration in the blood, indicating that the body’s oxygen transport volume was reduced in individuals with anemia, causing oxidative stress [34]. Oxidative stress is a critical factor in developing age-related chronic diseases caused by muscle wasting and inflammation [35]. Because of insufficient oxygen supply to the skeletal muscle, hypoxemia caused by anemia is thought to increase oxidative stress levels and cause sarcopenia. In addition, according to one previous study, a decrease in muscle mass causes changes in red blood cell mass, oxygen utilization rate [36]. Therefore, sarcopenia and anemia may be associated due to the interaction of physiological reactions.

Secondly, nutritional status can exacerbate sarcopenia and anemia [37,38], and the relationship between the two can be bidirectional. Anemia has been identified as a biomarker for weakness [39], and Hb levels reflect overall nutritional status [32]. Furthermore, low muscle mass is associated with frailty, weakness, and fatigue [40,41], which worsens with malnutrition [42,43]. According to this study, participants with sarcopenia had significantly lower intakes of the three major nutrients, carbohydrates, proteins, and fats, than those in the normal group. Malnutrition causes sarcopenia and anemia [37,38], and chronic sarcopenia can reduce Hb levels in the elderly.

Third, the level of C-reactive protein (CRP) in the blood is frequently elevated in elderly. Patients with sarcopenia have higher CRP levels than those healthy elderly [44], and inflammatory biomarkers significantly correlate with muscle damage, possibly leading to a more significant loss of muscle mass. In addition, CRP levels can predict iron reactivity in patients with anemia [45]. A high CRP level indicates an ongoing inflammatory process in the body, which affects iron availability, and reduces iron absorption [46]. Therefore, physical weakness due to acute or inflammatory reactions may have caused sarcopenia and anemia.

Fourth, cachexia is a syndrome characterized by marked weight loss, anorexia, asthenia, and anemia [47]. The main cause is cytokine excess, and other mediators are hormone imbalances such as testosterone and insulin-like growth factor-1 (IGF-1) deficiency, excess myostatin, and excess glucocorticoid [48]. These hormone and cytokine imbalances can cause anemia, fatigue, hypermetabolism, enhanced fat oxidation, and atrophy [49]. Sarcopenia and cachexia are two muscle-wasting disorders characterized by oxidative stress and inflammation, which means that regulating molecules are increased in expression (e.g., members of the ubiquitin–proteasome system, myostatin, apoptosis-inducing factors), whereas other factors (e.g., IGF-1) are down-regulated [50,51]. Therefore, due to the cytokine and hormone imbalance related to cachexia, the association between sarcopenia and anemia may have emerged.

Fifth, sarcopenia and anemia are affected by nutritional status and physical activity [52,53,54]. Lack of physical activity is associated with muscle mass and muscle loss, and resistance exercise training is the most widely used and has a positive effect on muscle strength and physical function [55]. In addition, low food intake and a monotonous diet are common in old age, which increases the opportunity for lack of nutrient intake [56]. In addition, studies have shown that lack of high-quality diet and intake of protein, antioxidant nutrients, and multiple unsaturated fatty acids are associated with decreased body function [57]. The results of this study also showed significant differences in energy intake and carbohydrate, protein, and fat intake in the sarcopenia group compared to normal. In addition, differences were also found in resistance and aerobic exercises in men. This difference is thought to have affected the results of this study. In particular, the association between sarcopenia and anemia according to sex was found in men, not women. As such, nutritional status and exercise are seen as variables that can have a significant impact on the two diseases.

As can be seen from the results of this study, the association between sex-based sarcopenia and anemia can not only optimize tailoring exercise intervention, prevention and management strategies based on sex differences, but also improve personalized health outcomes considering nutritional status and physical activity. Moreover, this study can provide insight into various sensitivities by exploring hormonal, genetic, and metabolic factors, and can pave the way for future research to uncover fundamental mechanisms by sex.

Summarizing the results of this study, sarcopenia was associated with anemia in the elderly, and this association is especially more significant in men. It is important to consider these confounding factors because SMI and Hb, the variables that determine sarcopenia and anemia, can be affected by various physical factors such as age, multiple metabolic factors, smoking and drinking, and exercise. These findings can play an important role as a basis for a sex-based therapeutic approach in clinical practice. However, further research is needed to explain the mechanisms for them.

Despite some significant findings, there were several limitations to evaluating the results of this study. First, nutritional data on micronutrient deficiency, including vitamin B12 and folic acid deficiency, one of the leading causes of anemia, are insufficient. Additionally, KNHANES failed to rule out drugs that could affect anemia and sarcopenia because there is no specific data on drug use and type. Therefore, it was difficult to consider their effects. Second, in this study, as anemia was defined solely by the Hb level, it was challenging to examine the type or cause of anemia. Therefore, further research is needed on variables that can affect anemia. Third, the small number of patients with severe sarcopenia or anemia in the KNHANES may have affected the analysis outcomes. However, given these data were obtained from the national population, it is not believed that a small number of individuals’ disturbance variables would have a major impact on the outcomes. In addition, despite these limitations, these data have the advantage of high response rates and accuracy as measured by professional medical staff. Fourth, there was a draw recall bias because the socio-statistical characteristics of the research population were gathered through questionnaires. However, this process was most likely eliminated randomly and was unlikely to have had a major influence on the study results. Fifth, this study used KNHANES data representing Korean population, but since it was data from 12 years ago, the results of this study cannot be said to be the same in the current population. However, this study is meaningful as a starting point for follow-up research. Sixth, because it represents the population of Korea, differences may appear in different races. Finally, while this study may help provide additional information about the nature of this relationship, it was a cross-sectional study that investigated sarcopenia and anemia simultaneously. Therefore, a temporal relationship could not be determined, and it was impossible to accurately determine the order of the underlying causes between the two factors. Therefore, caution should be taken when interpreting the results, and future longitudinal studies should be conducted to find a mechanism that can explain the association between the two. These findings could strengthen health care for patients with sarcopenia and anemia and provide literature support for future health education.

## 5. Conclusions

This study aimed to determine the association between sarcopenia and anemia in the elderly in Korea. Although influencing variables, such as personal lifestyle, exercise status, and nutritional intake, were considered, sarcopenia and anemia were independently associated. In particular, this association was significant in elderly men but not women.

## Figures and Tables

**Table 1:** Characteristics of participants stratified by anemia and sex.

Variables	Men			Women		
	Anemia (n = 104)	Normal (n = 1079)	*p*	Anemia (n = 244)	Normal (n = 1396)	*p*
Age (y)	73.13 ± 0.54	71.31 ± 0.16	0.001	74.45 ± 0.34	71.94 ± 0.16	<0.0001
Sarcopenia, % (n)	62.4 (67)	37.7 (408)	<0.0001	46.9 (107)	37.2 (511)	0.030
Weight (kg)	58.07 ± 1.11	63.44 ± 0.34	<0.0001	52.26 ± 0.74	55.26 ± 0.31	<0.0001
Height (cm)	163.49 ± 0.75	165.02 ± 0.20	0.049	149.94 ± 0.49	150.86 ± 0.19	0.077
BMI (kg/m^2^)	21.65 ± 0.33	23.25 ± 0.11	<0.0001	23.19 ± 0.27	24.24 ± 0.12	<0.0001
Education level, (%) (low/high)	81.1/18.9	75.3/24.7	0.049	20.7/79.3	18.3/81.7	0.031
Smoking status, (%) (current-/ex-/nonsmoker)	50.7/30.6/18.7	57.6/27.8/14.5	0.375	10.2/0.4/89.4	9.9/2.3/87.9	0.290
Drinking status (%) (current-/nondrinking)	65.7/34.3	72.0/28.0	0.215	25.1/74.9	38.4/61.6	<0.0001
Marital status, (%) (living with spouse)	91.4	91.4	0.989	62.0	53.2	0.030
Income (individual)			0.025			0.623
Q1 (lowest)	21.0	25.0		20.8	25.1	
Q2	18.2	25.9		27.7	24.7	
Q3	37.5	23.5		26.1	25.0	
Q4 (highest)	23.3	25.7		25.5	25.2	
Resistance exercise			0.525			0.549
Never	79.3	73.8		93.9	93.2	
1–3 days/wk.	10.0	11.9		2.5	3.8	
≥4 days/wk.	10.6	14.2		3.6	2.9	
Aerobic exercise (TWT)	69.59 ± 9.23	64.79 ± 3.10	0.632	68.98 ± 6.74	65.40 ± 3.01	0.614
Fasting glucose (mg/dL)	104.34 ± 2.73	103.67 ± 0.83	0.891	101.98 ± 1.75	104.45 ± 0.85	0.194
Total cholesterol	164.57 ± 3.25	184.52 ± 1.18	<0.0001	191.41 ± 2.24	203.98 ± 1.21	<0.0001
HDL-C	43.73 ± 1.25	45.38 ± 0.45	0.213	44.05 ± 0.74	46.79 ± 0.37	<0.0001
Triglyceride	106.08 ± 7.13	145.08 ± 3.37	<0.0001	131.34 ± 5.63	152.33 ± 3.29	0.002
Systolic BP (mmHg)	127.76 ± 1.78	130.68 ± 0.73	0.130	133.41 ± 1.51	132.80 ± 0.62	0.701
Diastolic BP (mmHg)	72.79 ± 1.06	77.73 ± 0.38	<0.0001	73.40 ± 0.74	77.16 ± 0.35	<0.0001
Waist circumference (cm)	80.09 ± 1.17	85.04 ± 0.32	<0.0001	80.61 ± 0.79	83.75 ± 0.34	<0.0001
High fasting glucose ^a^	48.4	43,8	0.419	51.6	56.2	0.577
Abdominal obesity ^b^	19.2	29.8	0.039	33.3	44.8	0.008
High triglyceride ^c^	16.0	34.9	0.001	29.3	39.6	0.009
High blood pressure ^d^	48.1	53.4	0.347	56.1	59.7	0.394
Low HDL-C ^e^	48.0	37.0	0.037	72.3	66.6	0.121
25(OH)D (ng/mL)	21.65 ± 1.04	21.84 ± 0.35	0.852	18.67 ± 0.57	18.62 ± 0.30	0.963
Energy intake (kcal)	1797.47 ± 65.17	1930.00 ± 28.30	0.053	1372.82 ± 39.60	1436.40 ± 16.77	0.143
Carbohydrate intake (g)	326.14 ± 11.43	334.51 ± 5.16	0.486	266.75 ± 7.91	276.54 ± 3.36	0.264
Protein intake (g)	58.94 ± 2.95	64.53 ± 1.27	0.790	42.51 ± 1.49	44.90 ± 0.72	0.145
Fat intake (g)	23.07 ± 1.47	27.79 ± 0.77	0.004	15.44 ± 0.80	17.47 ± 0.43	0.024
Iron intake (mg)	14.52 ± 1.29	15.77 ± 0.62	0.376	11.74 ± 1.10	12.056 ± 0.53	0.798
ASM (kg)	18.809 ± 0.29	19.75 ± 0.11	<0.0001	13.16 ± 0.16	13.51 ± 0.07	0.030
SMI (kg/m^2^)	6.75 ± 0.08	7.23 ± 0.03	<0.0001	5.84 ± 0.06	5.92 ± 0.02	0.166

Data were presented as the means ± SE or number (%). HDL-C; high density lipoprotein-cholesterol, ^a^ high fasting glucose level is defined as FBG ≥ 100 mg/dL; ^b^ abdominal obesity is defined as waist circumference >90 cm (men) or >85 cm (women); ^c^ high triglyceride level is defined as TG ≥ 150 mg/dL; ^d^ low HDL-C level is defined as HDL-C <40 mg/dL (men) or <50 mg/dL (women); ^e^ high blood pressure is defined as SBP ≥ 130 mmHg or DBP ≥ 85 mmHg.

**Table 2:** Characteristics of participants stratified by sarcopenia and sex.

Variables	Men			Women		
	Sarcopenia (n = 475)	Normal (n = 708)	*p*	Sarcopenia (n = 618)	Normal (n = 1022)	*p*
Age (y)	72.85 ± 0.25	70.71 ± 0.20	<0.0001	73.18 ± 0.25	71.79 ± 0.18	<0.0001
Sarcopenia, % (n)	12.8 (67)	5.1 (37)	<0.0001	18.8 (107)	13.4 (137)	0.030
Weight (kg)	56.89 ± 0.41	67.03 ± 0.36	<0.0001	50.08 ± 0.36	57.78 ± 0.33	<0.0001
Height (cm)	164.16 ± 0.30	165.39 ± 0.25	0.002	150.32 ± 0.31	150.96 ± 0.21	0.079
BMI (kg/m^2^)	21.08 ± 0.13	24.47 ± 0.11	<0.0001	22.13 ± 0.13	25.30 ± 0.12	<0.0001
Education level, (%) (low/high)	77.1/22.9	75.4/24.6	0.402	22.7/77.3	18.7/81.3	<0.0001
Smoking status, (%) (current-/ex-/nonsmoker)	59.3/26.8/13.9	55.6/28.9/15.5	0.542	13.0/1.4/85.7	8.0/2.4/89.7	0.012
Drinking status (%) (current-/nondrinking)	67.6/32.4	74.0/26.0	0.033	35.2/64.8	37.1/62.9	0.546
Marital status, (%) (living with spouse)	89.6	92.7	0.119	41.5	47.9	0.032
Income (individual)			0.020			0.731
Q1 (lowest)	23.5	25.4		23.0	25.4	
Q2	30.9	21.5		25.7	24.9	
Q3	22.4	26.1		24.7	25.4	
Q4 (highest)	23.1	27.0		26.6	24.4	
Resistance exercise			0.028			0.526
Never	79.3	71.0		94.1	92.9	
1–3 days/wk.	9.1	13.6		3.5	3.7	
≥4 days/wk.	11.7	14.5		2.4	3.4	
Aerobic exercise (TWT)	74.30 ± 4.81	59.16 ± 3.65	0.014	70.89 ± 4.36	62.83 ± 3.71	0.157
Fasting glucose (mg/dL)	103.45 ± 1.22	103.91 ± 1.02	0.769	102.40 ± 1.28	105.12 ± 0.94	0.078
Total cholesterol	180.53 ± 1.90	184.45 ± 1.46	0.102	204.21 ± 1.80	200.67 ± 1.33	0.110
HDL-C	46.91 ± 0.63	44.16 ± 0.50	<0.0001	47.18 ± 0.56	45.85 ± 0.42	0.053
Triglyceride	139.29 ± 4.91	143.62 ± 3.91	0.466	147.29 ± 3.90	150.21 ± 3.71	0.561
Systolic BP (mmHg)	130.01 ± 1.13	130.78 ± 0.80	0.586	132.34 ± 0.93	133.24 ± 0.72	0.421
Diastolic BP (mmHg)	76.64 ± 0.60	77.79 ± 0.44	0.109	75.38 ± 0.49	77.33 ± 0.44	0.002
Waist circumference (cm)	79.78 ± 0.49	87.84 ± 0.33	<0.0001	79.03 ± 0.42	85.93 ± 0.36	<0.0001
High fasting glucose ^a^	41.7	45.8	0.216	41.5	45.3	0.223
Abdominal obesity ^b^	12.6	39.8	<0.0001	23.3	54.4	<0.0001
High triglyceride ^c^	23.4	34.0	0.646	37.6	38.3	0.819
High blood pressure ^d^	51.7	53.8	0.583	57.3	60.3	0.331
Low HDL-C ^e^	32.4	41.6	0.007	67.6	58.3	0.063
25(OH)D (ng/mL)	21.27 ± 0.45	22.19 ± 0.45	0.098	18.15 ± 0.40	18.93 ± 0.35	0.103
Energy intake (kcal)	1778.95 ± 37.35	2011.69 ± 31.45	<0.0001	1345.73 ± 23.82	1477.60 ± 19.29	<0.0001
Carbohydrate intake (g)	314.47 ± 6.35	346.58 ± 5.89	<0.0001	259.04 ± 4.69	285.12 ± 3.90	0.001
Protein intake (g)	58.72 ± 1.76	67.60 ± 1.41	<0.0001	41.79 ± 1.00	46.25 ± 0.84	0.001
Fat intake (g)	23.76 ± 1.00	29.81 ± 0.96	<0.0001	16.38 ± 0.60	17.64 ± 0.50	0.102
Iron intake (mg)	14.87 ± 0.63	16.19 ± 0.61	0.241	10.08 ± 0.37	13.22 ± 0.73	<0.0001
ASM (kg)	17.34 ± 0.10	11.94 ± 0.07	<0.0001	11.94 ± 0.07	14.41 ± 0.06	<0.0001
SMI (kg/m^2^)	6.42 ± 0.03	7.70 ± 0.02	<0.0001	5.28 ± 0.02	6.31 ± 0.02	<0.0001

Data were presented as the means ± SE or number (%). HDL-C; high density lipoprotein-cholesterol, ^a^ high fasting glucose level is defined as FBG ≥ 100 mg/dL; ^b^ abdominal obesity is defined as waist circumference >90 cm (men) or >85 cm (women); ^c^ high triglyceride level is defined as TG ≥ 150 mg/dL; ^d^ low HDL-C level is defined as HDL-C <40 mg/dL (men) or <50 mg/dL (women); ^e^ high blood pressure is defined as SBP ≥ 130 mmHg or DBP ≥ 85 mmHg.

**Table 3:** Odds ratios for sarcopenia by anemia.

Model	Sarcopenia	OR (95% CI)	*p*
Model 1	Total	1.805 (1.364–2.388)	<0.0001
	Men	2.746 (1.740–4.334)	<0.0001
	Women	1.494 (1.045–2.138)	0.028
Model 2	Total	1.631 (1.228–2.166)	0.001
	Men	2.514 (1.585–3.988)	<0.0001
	Women	1.376 (0.950–1.992)	0.091
Model 3	Total	1.503 (1.098–2.058)	0.011
	Men	2.777 (1.545–4.991)	0.001
	Women	1.221 (0.833–1.788)	0.305
Model 4	Total	1.455 (1.064–1.989)	0.019
	Men	2.649 (1.475–4.755)	0.001
	Women	1.216 (0.827–1.788)	0.320

Reference category: participants with normal (non-anemia); Model 1 = crude; Model 2 = adjusted for age, sex; Model 3 = adjusted for variables in model 2 + BMI, education level, smoking, drinking, marital status, income level, high fasting glucose level, abdominal obesity, high triglyceride level, low HDL-C level, high blood pressure; Model 4 = adjusted for variables in model 3 + vitamin D level, resistance and aerobic exercise, energy, carbohydrate, protein, fat, and iron intake.

## Data Availability

All data were anonymized and can be downloaded from the website (https://knhanes.kdca.go.kr/knhanes (accessed on 20 June 2023)).
